# Factors influencing doctors’ counselling on patients’ lifestyle habits: a cohort study

**DOI:** 10.3399/bjgpopen18X101607

**Published:** 2018-09-19

**Authors:** Anna Sofia Viktoria Belfrage, Kjersti Støen Grotmol, Reidar Tyssen, Torbjørn Moum, Arnstein Finset, Karin Isaksson Rø, Lars Lien

**Affiliations:** 1 Psychologist and PhD student, Department of Behavioural Sciences in Medicine, Institute of Basic Medical Sciences, Faculty of Medicine, University of Oslo, Oslo, Norway; 2 Psychologist and PhD student, Norwegian National Advisory Unit on Concurrent Substance Abuse and Mental Health Disorders, Innlandet Hospital Trust, Brumunddal, Norway; 3 Researcher and Psychologist, Department of Behavioural Sciences in Medicine, Institute of Basic Medical Sciences, Faculty of Medicine, University of Oslo, Oslo, Norway; 4 Researcher and Psychologist, Regional Centre of Excellence in Palliative Care, Department of Oncology, Oslo University Hospital, Oslo, Norway; 5 Professor, Department of Behavioural Sciences in Medicine, Institute of Basic Medical Sciences, Faculty of Medicine, University of Oslo, Oslo, Norway; 6 Professor, Department of Behavioural Sciences in Medicine, Institute of Basic Medical Sciences, Faculty of Medicine, University of Oslo, Oslo, Norway; 7 Professor, Department of Behavioural Sciences in Medicine, Institute of Basic Medical Sciences, Faculty of Medicine, University of Oslo, Oslo, Norway; 8 Researcher, Department of Behavioural Sciences in Medicine, Institute of Basic Medical Sciences, Faculty of Medicine, University of Oslo, Oslo, Norway; 9 Professor, Faculty of Public Health, Inland Norway University of Applied Science, Elverum, Norway; 10 Professor, Norwegian National Advisory Unit on Concurrent Substance Abuse and Mental Health Disorders, Innlandet Hospital Trust, Brumunddal, Norway

## Abstract

**Background:**

Lifestyle changes are important for prevention and treatment of many common diseases, and doctors have an important role in the lifestyle counselling of patients. It is important to know more about factors influencing lifestyle counselling.

**Aim:**

To investigate the frequency of counselling about physical activity compared to that about alcohol habits; the impact of doctors’ own physical activity and alcohol habits on patient counselling about these lifestyle dimensions; and whether perceived mastery of clinical work or vulnerable personality have a confounding or moderating effect on these associations.

**Design & setting:**

In this nationwide cohort survey, a total of 978 doctors in Norway were surveyed by postal questionnaires in 1993/94 and 2014. The response rate was 562/978 (57%).

**Method:**

The outcome variables were questions on frequency of asking about alcohol and exercise habits. Explanatory variables were questions on doctors’ own exercise habits, drinking habits (using Alcohol Use Disorders Identification Test [AUDIT]), perceived mastery of clinical work, vulnerable personality, and specialty. Associations were studied by linear regression analysis.

**Results:**

Of the 526 responders, 307 (58%) reported asking usually/often about exercise habits, while *n* = 140/524 (27%) usually/often asked about alcohol habits. A doctor's own physical activity level was associated with frequency of asking about physical activity (unstandardised regression coefficient [*B*] = 0.07; 95% confidence intervals [CI] = 0.01 to 0.13). There were no significant associations between doctors' own lifestyle habits and counselling on alcohol habits. Doctors with low levels of vulnerability asked more frequently about physical activity, regardless of their own physical activity habits (*F* = 2.41, *P* = 0.048).

**Conclusion:**

Doctors’ own lifestyles influenced their preventive counselling about physical activity, but not about alcohol. Vulnerability moderated these effects, indicating the importance of early interventions to help doctors with a vulnerable personality to handle negative criticism from patients.

## How this fits in

Previous studies show that doctors’ own lifestyle habits have an impact on their counselling practices regarding lifestyle habits. Most importantly, this study shows that physicians tend to ask their patients less frequently about alcohol habits than about physical activity. Moreover, this study indicates that having a vulnerable personality moderates the effect of the doctors’ own physical activity when it comes to counselling on physical activity. Thus, helping doctors with a vulnerable personality to handle negative criticism from patients in a better way could lead to more well-functioning doctors as well as patients getting the counselling they need.

## Introduction

Lifestyle changes are essential for prevention and treatment of many common diseases, such as high blood pressure, overweight, diabetes, and substance abuse.^1–4^ Counselling about patients’ lifestyle habits is therefore important in medical consultations. Even if the long term beneficial medical effects of lifestyle counselling, such as lower mortality, are difficult to ascertain,^1^ a large study shows the positive effect of lifestyle counselling on patients' lifestyle habits.^2–6^ Still, addressing lifestyle habits can be difficult for many practicing doctors^7–9^ and more frequent counselling is needed. There can be a number of reasons why this is difficult. First, doctors' own lifestyles could be of importance; practitioners who smoke, do little exercise, or eat unhealthy food are less likely to counsel about these areas,^7^ while doctors with a healthy lifestyle counsel more often on lifestyle habits.^9–13^ Second, doctors’ sense of mastery of their work could be influential. Sense of mastery of work is similar to work self-efficacy; that is, the feeling of being able to handle demanding situations at work.^14^ Knowledge and self-efficacy regarding screening instruments were significant predictors of doctors’ use of screening for alcohol problems in a Norwegian study.^8^ Third, doctors’ personality could have an impact. A tendency to be uncomfortable in new situations or having difficulties handling negative criticism, which are components of a vulnerable personality,^15,16^ may have a negative impact on counselling about sensitive issues, among which lifestyle habits are likely to be included. While some patients appreciate lifestyle counselling, others can find it offending,^3^ and this uncertainty can be harder to cope with for a doctor with a vulnerable personality. Therefore, when studying the relationship between doctors’ lifestyle habits and the frequency of counselling patients about the same habits, it is essential to take the doctors’ sense of clinical mastery and their personality traits into account.

On this basis, this study aims to examine any effects of the doctors’ own lifestyle habits on the frequency of asking patients about those same habits. Two habits were chosen to study: physical activity as an example of a 'positive' lifestyle habit, and alcohol use as an example of a more 'negative' lifestyle habit. Moreover, the authors aim to study to what extent perceived mastery of work and/or a vulnerable personality influence the frequency of asking about those same lifestyle habits, and/or possibly moderate the association between the physician’s own lifestyle and frequency of asking about lifestyle habits.

In summary, it is hypothesised that even if the doctor is not physically active or has problematic drinking behaviour, having a high sense of mastery of their clinical work (meaning that the doctor feels comfortable in the clinical situation) could still induce the doctor to ask more frequently about physical activity or alcohol habits. In line with the same argument, it is hypothesised that even if a doctor is physically active or has healthy alcohol habits, high levels of vulnerable personality could induce the doctor to ask less frequently about physical activity or alcohol habits.

There is reason to believe that lifestyle counselling is handled differently in different specialties. GPs^17^ feel a greater responsibility to conduct general lifestyle counselling as part of a long term relationship with the patient, which is why this specialty is controlled for, comparing GPs and hospital medical physicians to other specialties. This study also controls for job stress, as a high degree of stress could potentially influence the doctors’ time for counselling during a consultation.

The primary object of the study is to investigate possible factors influencing doctor’s counselling on patients’ lifestyle habits. The research questions are: how frequently do doctors in different specialties counsel their patients about physical activity compared to alcohol habits? Are doctors’ own lifestyles associated with how often they counsel patients about physical activity and alcohol habits? Is having a sense of mastery of clinical work or a vulnerable personality associated with how often a doctor counsels their patients about physical activity and alcohol habits? Does having a sense of mastery of clinical work, or a vulnerable personality, have a moderating effect on possible associations between a doctor's own lifestyle, and counselling about the same lifestyle?

## Method

### Participants and study design

This study is based on data from the Longitudinal Study of Norwegian Medical Students and Doctors (NORDOC), which consists of two cohorts: the student cohort and the young doctor cohort. The study uses data from both the student cohort, consisting of all students who were enrolled in all four medical faculties in Norway in 1993 (*N* = 421; mean age 22 years, standard deviation [SD] 3.0; 53% female), and the young doctor cohort, consisting of all students who graduated from all four medical faculties in Norway in 1993/94 (*N* = 631; mean age 28 years, SD 2.8; 57% female).^18,19^ In 1993/94 all students were surveyed by postal questionnaires of about 30–40 pages and then followed up at six measurement points over a period of approximately 20 years (1993–2014). Several other studies on physicians’ health have been conducted with this data material.^19–26^ In this study, data are used from the baseline and from the last measurement point in 2014, at which point 57% (*n* = 562/978) of participants responded. The comparatively high response rate^27^ could be due to a substantial work in the beginning of the study in 1993/94, when researchers travelled to all medical schools in the country and personally informed medical students about the study. Thereafter, participants were reminded if they had not answered the survey, and at each follow-up were awarded a music CD as an incentive to respond. The two cohorts have been merged in the present study to get a bigger sample, which means that the medical student cohort is surveyed in the 15^th^ postgraduate year (PGY) in 2014, while the young doctor cohort is surveyed in the 20^th^ PGY. However, any stage-of-career effects are controlled for, as described below. Apart from vulnerability being measured at baseline, the study has a cross-sectional design.

### Dependent variables

The two dependent variables, counselling on physical activity and counselling on alcohol habits, are measured by one survey item each: 'How often do you talk with your patients about exercise habits/physical activity?' and 'How often do you talk with your patients about alcohol?' Responses were given on a 3-point ordinal scale: 1 = never/seldom, 2 = now and then, 3 = often/usually. The variable has been used and validated elsewhere.^28,29^

### Independent variables

#### Physical activity

Physical activity is measured by one survey item on doctors’ own physical activity: 'Do you usually conduct any form of exercise or work out, e.g. jogging, longer walks/skiing, body-building, cycling, swimming, playing football, tennis or similar?' Responses were on a 5-point ordinary scale: 1 = no, 2 = <1 day/week, 3 = 1–2 days/week, 4 = 3–4 days/week, 5 = 5–7 days/week. The variable has previously been validated.^30^

#### Alcohol habits

Alcohol habits are measured by a slightly modified version of AUDIT,^31^consisting of 10 items. Item 1 is coded: 0 = never, 1 = once a month or more seldom, 2 = 2–4 times a month, 3 = 2–4 times a week, 4 = every day or almost every day. Item 2 is coded as item 3 of the original AUDIT is: 'How often do you drink as much as 5 half bottles of beer (0.33cl), or a whole bottle of red or white wine, or a half bottle of liqueurs, or one quarter of a bottle of liquor?'. Responses are coded in the same way as item 1. The modified item 3 has been validated in previous NORDOC studies to measure hazardous drinking.^24,32^

#### Mastery of work

Perceived mastery of clinical work is measured by four items:^33^ 'I have sufficient knowledge and experience to do a good job as a physician'; 'I communicate without problems with patients and their next-of-kin'; 'I manage to establish collation with patients who are poor collaborators to begin with'; and 'I experience that I master the professional aspects that my work demands of me'. Responses are on a 7-point Likert scale from 1 = I agree to 7 = I don’t agree. Responses range from 4–28 (Cronbach’s α = 0.84).

#### Vulnerability

The vulnerability personality trait is measured by the vulnerability dimension of the Basic Character Inventory, originally constructed by Lazare *et al* ^34^ and modified by Torgersen.^15^ The previously validated^24,32^ vulnerability dimension — measuring emotional weakness, dependency, insecurity, and neuroticism — is based on nine items, each with a dichotomous response (agree/do not agree), giving a score range between 0–9 (low to high, [Cronbach’s α = 0.76]). This variable was measured in 1993/94.

#### Specialty

This study focuses on two specialties: GPs and hospital medical physicians. These are used as two dummy variables, GPs (*n* = 118) and hospital medical physicians (*n* = 339), with the reference category being 'other specialties'.

#### Job stress

Job stress is measured by a modified version of the Cooper Job Stress Questionnaire,^35^ including four dimensions: emotional pressure (Cronbach’s α = 0.86), fear of complaints and criticism (Cronbach’s α = 0.79), time pressure (Cronbach’s α = 0.72), and work–home interference (Cronbach’s α = 0.91).^36^ This measure has previously been validated.^20,25^ Responses are on a 5-point Likert scale from 1 = not at all to 5 = very much.

#### Stage of career (cohorts)

Different stages of the doctors’ careers could play a role, thus stage-of-career effects are controlled for in the analytical models.

### Statistical analyses

Paired sample *t*-tests were run to determine whether differences in how often doctors asked about physical activity and alcohol habits were significant.

Analyses of covariance (ANCOVAs) were conducted to reveal possible associations, using sex and specialty as factors and continuous variables as covariates. Adjusted analyses were performed in two blocks; to initially study the effects of a doctor's own lifestyle habits on counselling (block I), and to control for other possible confounders or moderators (block II). All explanatory variables were therefore controlled for each other, but in separate steps. Block I included age, sex, and doctors' own lifestyle habits. In block II, all variables were entered from block I with *P*<0.1, as well as vulnerable personality, perceived mastery of work, and specialty (stage-of-career was included as a variable, to rule out any stage-of-career effects).

To check for any moderating effects on counselling, the authors added two-way interactions between lifestyle variables and each of the other independent variables (possible moderators). Each interaction term was added separately (that is, one at a time) in the ANCOVAs. Tests of interaction were performed with continuously measured variables.

## Results

### Frequency of counselling

**Figure 1.**
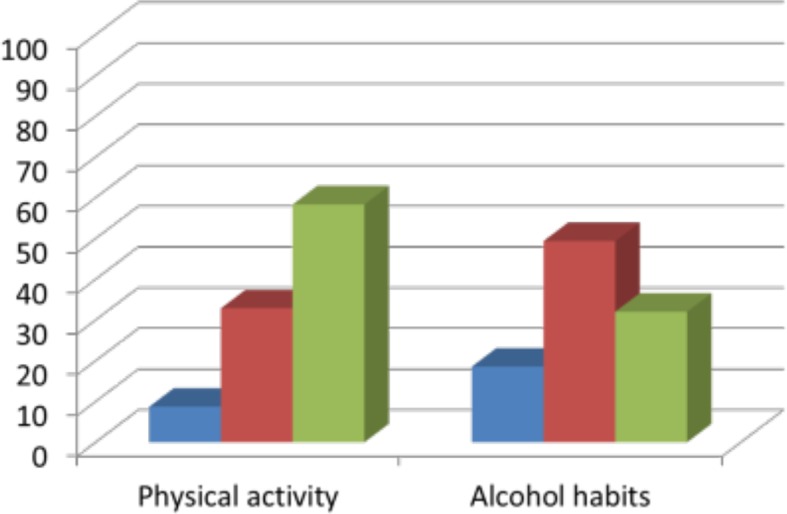
Frequency (%) of counselling about physical activity (N = 526) and alcohol habits (N = 524) in 2014 for all doctors

Fifty-eight percent of doctors counselled usually/often about physical activity and 27% counselled usually/often about alcohol habits (*P*<0.001). (Figure 1.) A higher percentage of GPs counselled usually/often about physical activity (85%) compared with frequency of counselling about alcohol habits (31%), and hospital medical physicians counselled patients more frequently about their physical activity (51%) than about their alcohol habits (26%).

#### Description of lifestyle habits among the doctors

**Figure 2.**
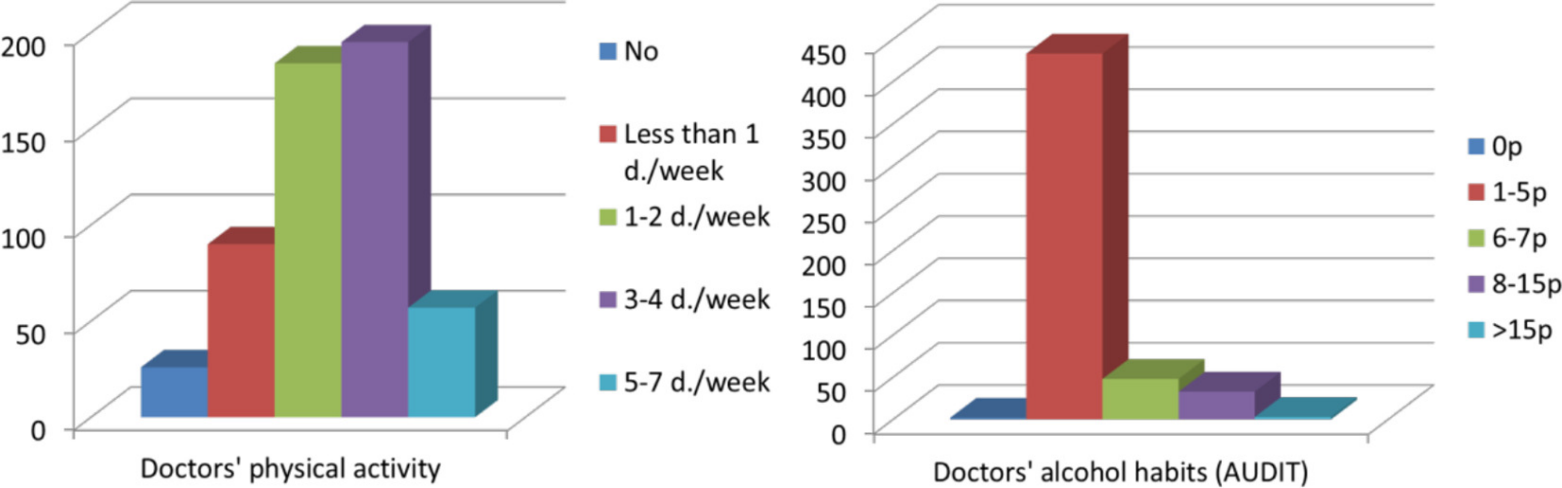
Doctors’ own physical activity level and alcohol habits in 2014.

Frequency of physical activity and alcohol habits (AUDIT score) among the doctors in 2014 is shown in Figure 2.

### Factors associated with doctors' counselling on patients’ physical activity

#### Associations between doctors' own physical activity and counselling about physical activity

There is a significant positive association between doctors’ own physical activity and how often they asked their patients about physical activity (*B* = 0.10; 95% CI = 0.04 to 0.15) (Table 1, block I). When other factors are controlled for in block II, vulnerability (*B* = -0.04; CI = -0.06 to -0.01) and type of specialty (being a GP versus being a hospital medical physician [*B* = 0.42; CI = 0.22 to 0.62]) are significantly associated with counselling about physical activity, in addition to doctors’ own activity (*B* = 0.07; CI = 0.01 to 0.13). Further analyses show that vulnerability is a confounder of the association between a doctor's own physical activity and counselling on physical activity, while specialty is not.

**Table 1.** Factors associated with counselling about physical activity in 2014

				Adjusted		
	**Unadjusted**		**Block I^a^**		**Block II^b^**	
	B	95% CI	B	95% CI	B	95% CI
**Demographic factors**						
Age	0.003	-0.01 to 0.02	-0.003	-0.01 to 0.02	-0.002	-0.02 to 0.015
Sex	-0.02	-0.14 to 0.09	-0.01	-0.13 to 0.10	-0.02	-0.15 to 0.10
**Own lifestyle habits**						
Own physical activity	0.10^d^	0.04 to 0. 16	0.10^d^	0.04 to 0.15	0.07^e^	0.01 to 0.13
Own alcohol habits	-0.001	-0.02 to 0.02				
**Confounders/moderators**						
Clinical mastery	0.02^e^	0.001 to 0.04			0.01	-0.01 to 0.03
Vulnerability	-0.04^d^	-0.07 to -0.01			-0.04^e^	-0.06 to -0.01
GP^c^	0.44^f^	0.31 to 0.57			0.42^f^	0.22 to 0.62
Hospital medical physician^c^	-0.28^f^	-0.40 to -0.17			-0.01	-0.19 to 0.16
Job stress	0.003	-0.002 to 0.01				
Cohort	-0.02	-0.13 to 0.09				

^a^Block I = the result of adjusted analysis on associations between own lifestyle habits and counselling. ^b^Block II = the result of adjusted analysis on associations between own lifestyle habits and counselling, controlled for possible other confounders/moderators ^c^Reference category = other specialty. ^d^ *P*<0.01. ^e^ *P≤﻿*0.05. ^f^ *P*<0.001.

B = unstandardised beta. CI = confidence intervals.

#### Moderators of the effect of doctors’ own physical activity on their counselling regarding physical activity

**Figure 3.**
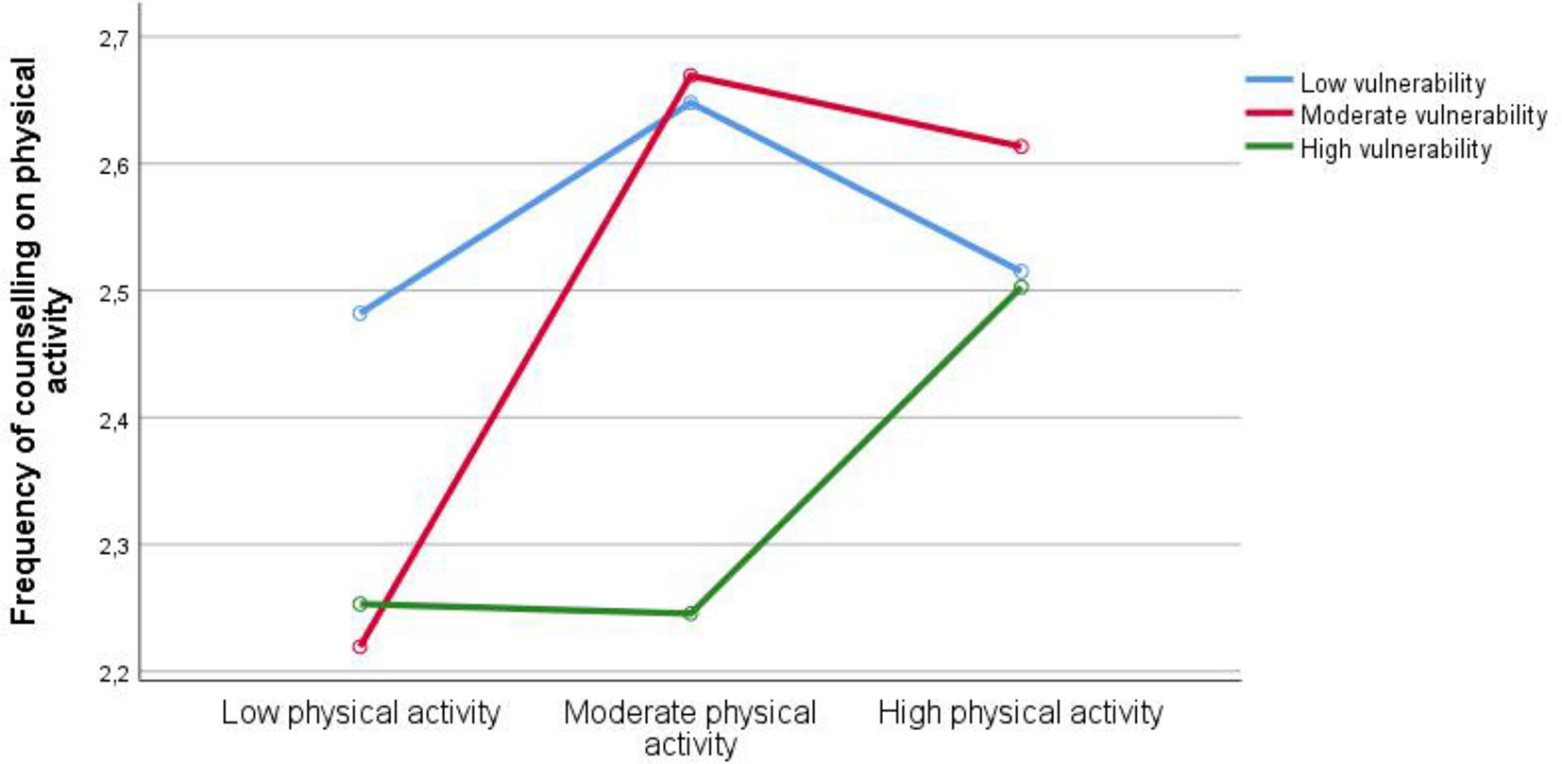
Interaction of vulnerability on own physical activity when counselling on physical activity

Interaction analyses yielded no significant interactions between variables measured continuously. Additional analyses did, however, reveal a significant interaction effect between low, moderate, and high vulnerability and low, moderate, and high physical activity (*F* = 2.41, *P* = 0.048). It was found that those with high vulnerability needed a high level of physical activity themselves to counsel as frequently on physical activity as the other doctors, while doctors with moderate vulnerability needed only a moderate level of physical activity of their own to counsel as frequently as other doctors on physical activity. Doctors with a low level of vulnerability asked frequently about physical activity regardless of their own physical activity level. (Figure 3).

### Factors associated with doctors' counselling on patients’ alcohol habits

#### Associations between doctors' own alcohol habits and counselling about alcohol

There are no significant associations between doctors’ own alcohol habits and how often they ask their patients about alcohol habits, nor between the doctor's sex and their counselling on alcohol habits. GPs (*B* = 0.27; CI = 0.08 to 0.47) asked more frequently about alcohol habits than other specialties, with no controlled difference between hospital medical physicians and other specialties (Table 2).

**Table 2.** Factors associated with counselling about alcohol habits in 2014

				Adjusted		
	**Unadjusted**		**Block I^a^**		**Block II^b^**	
	B	95% CI	B	95% CI	B	95% CI
**Demographic factors**						
Age, years	0.01	-0.003 to 0.03	0.01	-0.003 to 0.003	0.01	-0.002 to 0.03
Sex	0.01	-0.11 to 0.12	0.004	-0.12 to 0.12	-0.006	-0.13 to 0.11
**Own lifestyle habits**						
Own physical activity	0.05	-0.01 to 0.11	0.05	-0.01 to 0.11		
Own alcohol habits	-0.01	-0.03 to 0.02				
**Confounders/moderators**						
Clinical mastery	<0.001	-0.02 to 0.02				
Vulnerability	0.002	-0.03 to 0.03				
GP^c^	0.25^d^	0.11 to 0.39			0.27^d^	0.08 to 0.47
Hospital medical physician^c^	-0.28^e^	-0.40 to -0.17			0.04^e^	-0.13 to 0.21
Job stress	0.002	-0.003 to 0.01				
Cohort	0.02	-0.10 to 0.14				

^a^Block I = the result of adjusted analysis on associations between own lifestyle habits and counselling. ^b^Block II = the result of adjusted analysis on associations between own lifestyle habits and counselling, controlled for possible other confounders/moderators. ^c^Reference category: other specialty. ^d^ *P*<0.01.^e^ *P*<0.001. B = unstandardised beta. CI = confidence intervals.

#### Moderators of the effect of doctors’ own alcohol habits on their counselling regarding alcohol

ANCOVA analysis yielded no significant interaction (moderating) effect of perceived mastery or vulnerability on the association between doctors’ own drinking habits and counselling on alcohol.

## Discussion

### Summary

In line with the hypothesis, this study found that doctors counselled more often on physical activity than on alcohol habits (58% versus 27% respectively).

The more physically active doctors were, the more they counselled on physical activity. No association was found between doctors’ own alcohol habits and frequency of counselling on alcohol.

Vulnerability had a moderating effect on the association between doctors’ own physical activity and frequency of counselling patients about physical activity. Doctors with a high vulnerable personality trait score counselled more frequently about physical activity when they were highly physically active themselves, while doctors with a moderate vulnerability score needed only a moderate level of physical activity to counsel with the same frequency. For those reporting low levels of vulnerable personality traits, there was no association between their own physical activity and the frequency of asking about physical activity; these doctors would counsel at a high frequency about physical activity regardless of their own activity level. This study found no moderating effect of perceived mastery.

### Strengths and limitations

Strengths of this study are the nationwide sample and the relatively high response rates for this group. A range of possible predictors were not controlled for, such as the amount of time doctors had with each patient, their communication skills, or patient-specific factors. It would also have been interesting to have more data on practice venues, as city-based and rural practitioners could have different relationships to their patients that could influence counselling on lifestyle habits.

The relatively high response rate could be due to use of postal surveys, which are believed to give a higher response rate than alternate formats, such as internet surveys.^37^ With a response rate of 57% there is, however, risk of attrition bias, which limits the implications of this study. It is not known if those that did not respond to the survey more or less vulnerable, ask more or less frequently about alcohol habits, or have a more or less healthy lifestyle. There was little variation in the level of alcohol consumption in the group of doctors participating. This could make it difficult to study associations between the level of a doctor's own drinking habits and counselling for alcohol consumption. The interaction effect between vulnerability and physical activity on counselling on physical activity is weak and should be interpreted with caution. Besides, the outcome variables are on a 3-point Likert scale in which the middle alternative is 'now and then', which can be interpreted as not very often *or* occasionally. The lack of response options between 'now and then' and the other two, could bias the results. Furthermore, no objective measure is available to assess how frequently doctors do this in practice.

### Comparison with existing literature

In line with previous studies,^9,11,29^ an association was found between doctors' own physical activity and counselling about physical activity, partly moderated by vulnerability. This weak association could be explained by Norway being a country where the relationship between doctor and patient is more equal.^17,38^ Not counselling on physical activity when the doctor is physically active could be a sign of the doctor’s self-reflexivity (the ability to reflect on their professional role, and the potential influence of contextual factors)^39^ when bringing up sensitive issues,^40^ in this case, reflecting over the possibility that the patient might perceive counselling as showing superiority, when a physically fit doctor brings up the subject of physical activity with a physically inactive patient.

Despite a decrease in alcohol consumption among Norwegian doctors^41^ and an increase among the Norwegian population between 2000–2010,^42^ doctors still do not ask more often about alcohol habits. Thus, when it comes to frequency of counselling on alcohol habits, doctors’ own alcohol habits do not seem to play a role determining why doctors don’t ask about this issue. Other studies report that doctors find it time consuming, distracting, and even awkward to bring up the subject of alcohol habits.^43,44^ Asking about smoking habits is apparently not as hard for doctors as asking about alcohol habits.^43^ This could partly have to do with privacy surrounding alcohol use, and some practitioners feel that they lack the communication skills necessary for the task.^43,44^

This study shows that vulnerability is a moderating factor, and could indicate that doctors low in vulnerability personality traits are less sensitive to other factors (such as own lifestyle habits, or fear of offending their patients) when it comes to carrying out standard counselling. In an earlier study, the authors found that vulnerability (which includes difficulties in handling negative criticism and a belief that others do things better than oneself)^16^ was a long-term predictor of stable low sense of mastery of clinical work among Norwegian doctors.^45^ Presumably, the explanation could be a long-term pattern of avoiding challenging situations with patients, thereby avoiding being criticised.^45^ Less avoidance of challenges is, in turn, associated with higher professional resilience,^46^ as are an increased sense of self-worth and leisure activities.^47^ Resilience workshops, small group problem-solving, reflection, and cognitive behavioural training are some interventions shown to improve professional resilience,^48^ and could potentially influence the frequency of counselling on lifestyle habits in a positive direction.

### Implications for research and practice

The combination of having high vulnerability and low physical activity is associated with a marked reduction in the frequency with which a doctor asks about physical activity. When it comes to asking about alcohol, it is 'normal' not to ask about it. Studies show that interventions to improve GPs counselling on alcohol and other lifestyle habits have had limited effect.^49,50^ Still, one qualitative Norwegian study indicates that 'pragmatic case finding' (where the patient’s alcohol habits are addressed as part of other clinical problems where alcohol habits may have an effect) is a better alternative than screening instruments or brief interventions when it comes to alcohol counselling.^51^ Perhaps, if asking about alcohol becomes more common, it might be possible to find a similar pattern of associations where vulnerable doctors who drink more alcohol will still not ask about alcohol, while others will. Future research needs to both study this hypothesis and further investigate which factors are barriers to counselling on alcohol habits.

Preventive counselling is an area with an increasing degree of focus. New guidelines underline the importance of counselling for lifestyle. GPs have had an important role in such counselling, but the trend that has been seen previously in other health systems, like the NHS,^52^ towards practice nurses and other staff taking over or complementing doctors in these tasks now seems to be becoming more commonplace in Scandinavia as well.^53^ A newly published study from Sweden shows that nurses follow up new guidelines on preventive practices to a wider extent than doctors do.^53^ Doctors increased their counselling about physical activity to a certain degree after the new guidelines, whereas no change was found in relation to alcohol counselling. To assess the total amount of preventive counselling that patients receive, it would thus be relevant to study the sum of preventive counselling from doctors, nurses, and other professions in primary care.

There is an ongoing discussion on whether personality is a trait or a state. Modern studies indicate that personality is a changeable state depending on what situations or challenges an individual encounters.^54,55^ The findings indicate that helping doctors with a vulnerable personality to handle negative criticism in a better way, for example, through guidance in exposing oneself to challenging situations and practicing in how to handle being criticised, could lead to the development of professional resilience^46^ and more well-functioning doctors, as well as patients getting the counselling they need.

Future research might broaden its approach by focusing both on quantity (the frequency of implementing screening tools)﻿ and on quality, by helping practitioners feel comfortable in bringing up sensitive issues in a non-confrontational way (for example, inspired by motivational interviewing) and helping practitioners develop self-reflexivity in apparently challenging situations.
